# Sentinel Hospital-Based Surveillance for Assessment of Burden of Rotavirus Gastroenteritis in Children in Pakistan

**DOI:** 10.1371/journal.pone.0108221

**Published:** 2014-10-08

**Authors:** Abdul Momin Kazi, Gohar Javed Warraich, Shahida Qureshi, Huma Qureshi, Muhammad Mubashir Ahmad Khan, Anita Kaniz Mehdi Zaidi, and members of the Pakistan Rotavirus Study Group

**Affiliations:** 1 Aga Khan University, Karachi, Pakistan; 2 Pakistan Medical Research Council, Islamabad, Pakistan; Johns Hopkins Bloomberg School of Public Health, United States of America

## Abstract

**Objectives:**

To determine the burden and molecular epidemiology of rotavirus gastroenteritis in children hospitalized with severe acute watery diarrhea in Pakistan prior to introduction of rotavirus vaccine.

**Methods:**

A cross-sectional study was carried out over a period of two years from 2006 – 2008 at five sentinel hospitals in the cities of Karachi, Lahore, Rawalpindi, and Peshawar. Stool samples collected from children under five years of age hospitalized with severe acute watery diarrhea were tested for rotavirus antigen via enzyme immunoassay (EIA) (IDEA REF K6020 Oxoid Ltd (Ely), Cambridge, United Kingdom). A subset of EIA positive stool samples were further processed for genotyping.

**Results:**

6679 children were enrolled and stool specimens of 2039 (30.5%) were positive for rotavirus. Rotavirus positivity ranged from 16.3% to 39.4% in the 5 hospitals with highest positivity in Lahore. 1241 (61%) of all rotavirus cases were in infants under one year of age. Among the strains examined for G-serotypes, the occurrence of G1, G2, G9 and G4 strains was found to be 28%, 24%, 14% and 13%, respectively. Among P-types, the most commonly occurring strains were P6 (31.5%) followed by P8 (20%) and P4 (12%). Prevalent rotavirus genotype in hospitalized children of severe diarrhea were G1P[8] 11.6% (69/593), followed by G2P[4] 10.4% (62/593), and G4P[6] 10.1% (60/593).

**Conclusions:**

Approximately one third of children hospitalized with severe gastroenteritis in urban centers in Pakistan have rotavirus. Introduction of rotavirus vaccine in Pakistan's national immunization program could prevent many severe episodes and diarrheal deaths.

## Introduction

According to global estimates in 2011, over 700,000 children die annually from diarrheal diseases [1], [2]. Of these rotavirus (RV) is responsible for an estimated 197,000 diarrhea deaths [2], [3]. While global diarrheal mortality has fallen with improvements in hygiene, sanitation and an increase in public health education, RV continues to be the most important cause of severe diarrhea and dehydration in young children the world over [4]–[6]. In Pakistan, a country with the fourth highest burden of child mortality, diarrheal diseases remain major killers [1], [6]. Although some burden data on the role of RV as a cause of severe diarrhea in Pakistan are available, there is a lack of systematically collected data from multiple hospital settings across the country with careful description of clinical syndromes representing the most severe diarrhea which can be taken as a proxy for deaths, and inform policy makers on the importance of RV as a cause of child deaths [7]–[14].

There are currently two licensed RV vaccines in widespread use, Rotarix (GlaxoSmithKine) and Rotateq (Merck). In 2009 the World Health Organization (WHO) recommended that RV vaccines be included in all national immunization programs [15]. Countries that have introduced RV vaccines have shown a substantial decline in diarrhea-related hospital admissions and deaths [16], [17].

RV is a double stranded RNA virus belonging to the retroviridiae family [18]. It possesses neutralizing epitopes in the outer capsid of its three concentric shells [19]. Two of the 11 segment genome code the proteins, VP4 – the P protein and the glycosylated VP7 – the G protein [19], [20]. G and P proteins comprise the binomial system of RV strain classification. The molecular epidemiology of RV keeps changing. The strains G1P [8], G2P [4], G3P [8], and G4P [8] accounted for over 90% of the world's disease burden, but these strains are found in only 68% of Asian diarrheal cases [21], [22].

Before introduction of the RV vaccination in national immunization programs it is important that the baseline burden of disease is well characterized so that the impact of introduction of vaccine can be evaluated. It is also important to monitor genotypic distribution since circulating strain types will vary over time and there is the potential for emergence of new strains following the introduction of RV vaccine.

## Methods

### Surveillance sites

**Figure 1:**
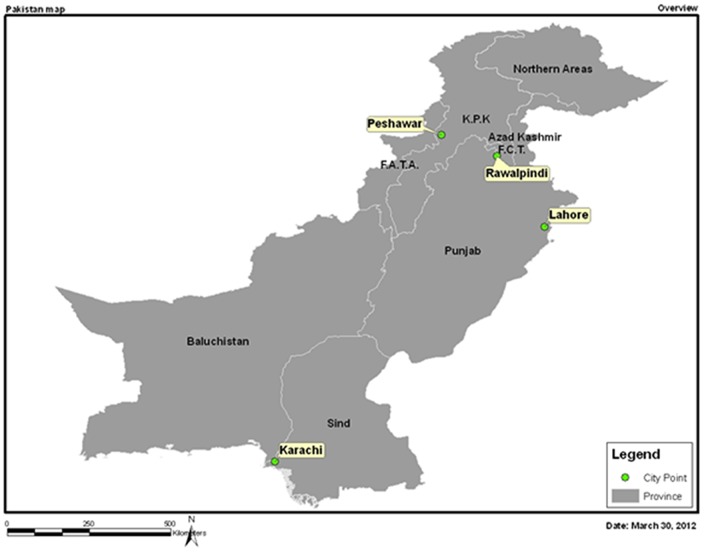
Geographic distribution of the five sentinel sites participating in rotavirus surveillance in Pakistan from 2006–2008.

The study was conducted at five sentinel hospitals because of their well-established pediatric care protocols and willingness to participate in the surveillance study. Samples were collected at: National Institute of Child Health (NICH) and Kharadar General Hospital (KGH) in Karachi; Lahore Children's Hospital (LCH) in Lahore; Mercy Hospital, Peshawar Medical College (MHPMC) in Peshawar and Rawalpindi General Hospital (RGH) in Rawalpindi (Figure 1).

Karachi is the largest metropolitan and principal port city of Pakistan. It is situated in the southernmost part of Pakistan: two sentinel sites – National Institute of Child Health and Kharadar General Hospital are located here. NICH is a 320 bed tertiary care public sector hospital providing care to children in Karachi with a dedicated diarrhea treatment unit. KGH is a 200 bed tertiary care hospital located in old city area of Karachi and run by a private charitable trust. The city of Rawalpindi is the twin city of the capital – Islamabad, located in the northern most part of the province of Punjab. Its public sector hospital – Rawalpindi General Hospital (now called Benazir Shaheed Hospital) is a 626-bed teaching hospital, with dedicated pediatric unit including diarrhea ward. South-east of Rawalpindi is Lahore, one of the most densely populated urban centers of the world. Lahore's Children Hospital, a 350 bed teaching hospital run by the public sector is the largest tertiary care hospital for children in the country. The city of Peshawar lies in Khyber Pakhtunkhwa province close to the western border and Federally Administrated Tribal Areas of Pakistan associated with Peshawar Medical College is run by a private charitable trust mainly serving refugee populations that are internally displaced or from Afghanistan. It has a dedicated diarrhea treatment unit.

### Case Definition

A suspected case of RV diarrhea was defined as a child under-five years of age who was hospitalized for gastroenteritis as the primary illness and had watery diarrhea of less than 7 days duration with severe dehydration as classified by WHO's Integrated Management of Childhood Illnesses (IMCI). WHO IMCI handbook defines severe dehydration for children less than five years old as a child having two of the following signs: lethargy or unconsciousness, sunken eyes, poor oral intake and/or skin pinch going back very slowly [23].

### Data collection

Clinical, demographic and laboratory details were recorded in a RV diarrhea case report form for all patients who met the case definition. Clinical information included mode of admission, duration of hospital stay, and history of diarrhea, fever and vomiting. Written informed consent was obtained from the parents and guardians of all study participants. The study protocol and the consent procedure was reviewed and approved by the Aga Khan University's Ethical Review Committee and Western Institutional Review Board (through the study sponsor, Program for Appropriate Technology in Health (PATH).

### Rotavirus antigen detection

After training of personnel included in the study, stool testing was done locally at the laboratory of the sentinel hospital where samples were collected. 6679 stool specimens were processed for the RV antigen detection by using IDEA REF K6020 (Oxoid Ltd (Ely), Cambridge, United Kingdom) according to manufacturer's instructions. Ten percent of analyzed samples were randomly selected and sent to Aga Khan University for quality control (i.e., every 10^th^ sample).

### G and P genotyping

All EIA RV positive stool samples were transported monthly at 20°C to AKU. A subset of 29% RV positive samples was then further processed for genotyping at the Infectious Disease Research Laboratory (IDRL) of the Department of Paediatrics and Child Health, AKU. The subset of RV positive stools selected for further characterization had contributions from all participating hospitals. Samples for genotyping were selected to be representative of all sites, seasons, and age strata. Funding constraints limited genotyping of all strains. RNA was extracted from RV positive stool suspensions by trizol method: Stool suspension was prepared with an equal volume of water. RNA fraction partitioned into the clear, upper aqueous phase. The phase separation and precipitation was facilitated by trizol, chloroform and isopropyl alcohol. Known positive controls and negative controls were used to validate the PCR assay. The strain specific primers used had sequence described by Gentsch et al[24]. A 2% Agarose Gel Electrophoresis was performed and 123 bp ladder marker was used as a reference for the VP4 and VP7 amplified products, gels were stained with Ethidium Bromide and documented under UV light in Gel Doc imager.

## Results

### Patients with Acute Diarrhea

Numbers of patients with acute watery diarrhea fulfilling eligibility criteria admitted during the study period from April 2006 to May 2008 were: 23,212 in LCH (Lahore), 2,028 in RGH (Rawalpindi), 937 in MHPMC (Peshawar), 2,417 in NICH (Karachi) and 1,991 in KGH (Karachi). A total of 6679 hospitalized children submitted a stool specimen for analysis. Of these, 26% were from LCH (Lahore), 24% from RGH (Rawalpindi), 12% stool specimens were collected from MHPMC (Peshawar), and 21% and 17% from NICH & KGH (Karachi), respectively.

### Acute Diarrhea Cases

**Figure 2:**
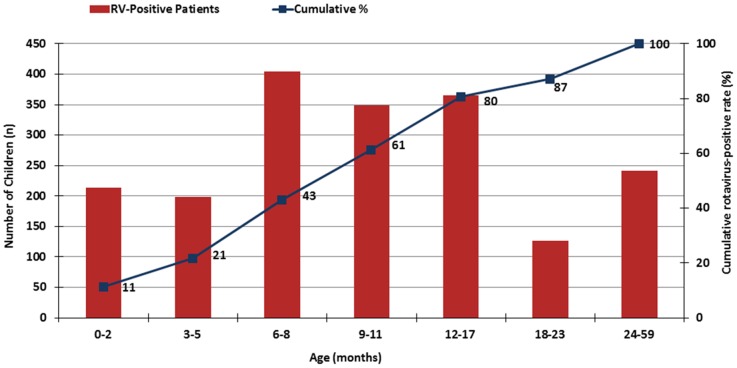
Cumulative age distribution of children less than 5 years of age admitted with severe diarrhea at five sentinel hospitals in Pakistan, 2006–2008. Bars represent number of children with rotavirus- positive diarrhea. Points represent cumulative rate (%) of rotavirus- positive gastroenteritis.

Of the total 6679 stool specimen collected, 30.5% (2039) tested ELISA positive for RV. The median age of RV cases was 10 months and 58.7% (1196) cases of children with RV positive stool samples were male. The age distribution among RV positive cases was 60.9% (1241) children <12 months, 26% (530) children between 12 to 23 months, and 13.1% (268) children between 24 to 59 months (see Table 1 and Figure 2). Site specific data (not shown), revealed RV positivity varied from 16.3% in NICH (Karachi) to 39.4% in LCH (Lahore). At the remaining sites, RV positive cases were 29.5% in KGH (Karachi), 30.9% in RGH (Rawalpindi) and 37.2% in MHMPC (Peshawar).

**Table 1:** Comparison of clinical features of children under 5 years of age with rotavirus positive gastroenteritis and rotavirus negative gastroenteritis admitted in five hospitals in Pakistan from 2006 – 2008.

Characteristics	RV +ve	RV –ve	Total	
	(n = 2039)	(n = 4640)	(n = 6679)	P-Value
	no. (%)	no (%)	no. (%)	
**Age (months)**				
0–11	1241(60.9)	2429(52.3)	3670(54.9)	<0.01
12–23	530(26)	1204(25.9)	1734(26)	
24–59	268(13.1)	1007(21.7)	1275(19.1)	
0–59	2039	4640	6679	
**Sex**				
Male	1196(58.7)	2613(56.3)	3809(57)	0.075
Female	843(41.3)	2027(43.7)	2870(43)	
Total	2039	4640	6679	
**Fever (> 99°F) a**				
Yes	1065(52.8)	2759(59.9)	3824(57.7)	<0.01
No	953(47.2)	1845(40.1)	2798(42.3)	
Total	2018	4604	6622	
**Vomiting a**				
Yes	1165(58.6)	2855(62.9)	4020(61.6)	<0.01
No	824(41.4)	1686(37.1)	2510(38.4)	
Total	1989	4541	6530	
Frequency of diarrhea*	10.4±4.3	10.3±4.1	10.3±4.2	0.09
Duration of diarrhea (in days)*	2.8±2.0	2.9±2.6	2.9±2.5	0.03

a Total do not add up to sum to missing data.

* Mean±SD.

### Clinical Features

The clinical features of RV positive and RV negative gastroenteritis are described in Table 1. There was no difference between the frequency of diarrheal stools between RV positive and RV negative gastroenteritis cases. Duration of diarrhea was slightly prolonged in RV negative gastroenteritis (2.9±2.6 days) as compared to RV positive gastroenteritis (2.8±2.0 days). RV negative gastroenteritis was more commonly accompanied by vomiting than RV positive gastroenteritis, 62.9% and 58.6%, respectively. Similarly RV negative gastroenteritis was also more likely to be present with fever (59.9%) as opposed to RV positive gastroenteritis (52.8%).

### Rotavirus Seasonality

**Figure 3:**
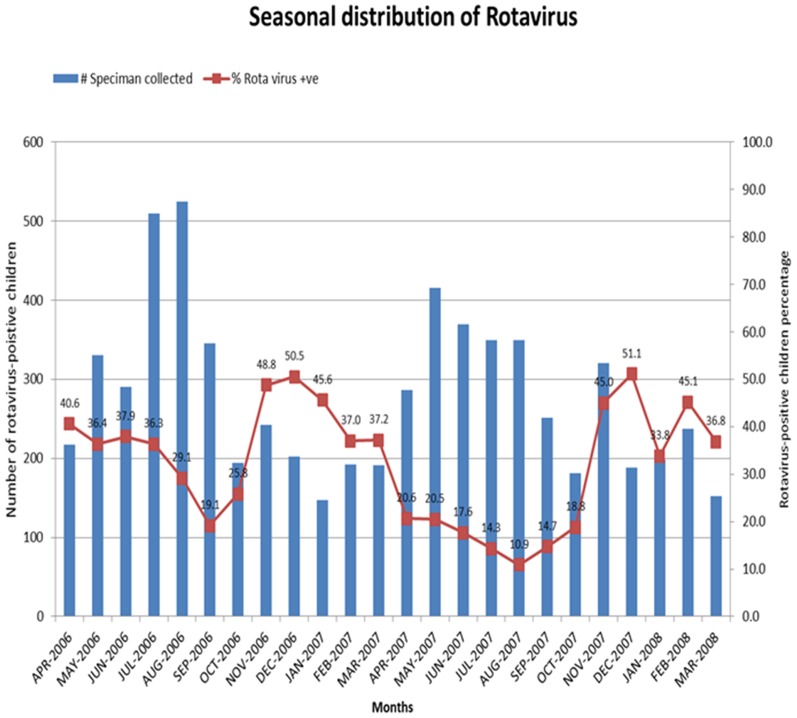
Distribution of rotavirus positive gastroenteritis by age in months at five sentinel hospitals in Pakistan from 2006–2008.

RV hospitalization was seen throughout the year over the period of the two years of surveillance, but peaked from November through to March (Figure 3).

### Rotavirus Genotyping

Genotyping analyses were carried out to determine the type and distribution of RV strains. Out of 2039RV positive stool samples, 593 (29.1%) were processed for genotyping and characterization for both G(VP7) and P(VP4) types using multiplex RT-PCR. Among the G-types examined, the occurrence of G1, G2, G9 and G4 strains was found to be 28%, 24%, 14% and 13%, respectively. Among P-types, the most commonly occurring strains were P[6] (31.5%) followed by P[8] (20%) and P[4] (12%). Prevalent RV genotype in our hospitalized children having severe diarrhea were G1P[8] 11.6% (69/593), followed by G2P[4] 10.4% (62/593), and G4P[6] 10.1% (60/593). Other G–P combinations were G1P[6] (5.4%, 32/539), G2P[6] (4.2%, 25/593), G9P[6] (4.0%, 24/593) and G9P[8] (3%, 18/593). Table 2 describes the genotyping results. Uncommon strains found in the study subjects were G2P[11], G2P[8] and G1P[9].

**Table 2:** Distribution of genotype of rotavirus strains determined in children less than 5 years of age admitted with acute gastroenteritis in Pakistan, 2006–2008.

P-type	G-type							
	G1(%)	G2(%)	G4(%)	G9(%)	G12(%)	Mixed(%)	UT(%)	Total (%)
**P[4]**	4(0.67)	62(10.4)	-	2(0.33)	-	6(1.01)	2(0.33)	76(12.8)
**P[6]**	32(5.39)	25(4.2)	60(10.1)	24(4.04)	2(0.33)	13(2.19)	31(5.22)	187(31.5)
**P[8]**	69(11.6)	6(1.01)	3(0.5)	18(3.03)	1(0.16)	6(1.01)	17(2.86)	120(20.2)
**P[9]**	2(0.33)	-	-	-	-	-	-	2(0.3)
**P[11]**	4(0.67)	9(1.5)	2(0.33)	5(0.84)	-	1(0.16)	5(0.84)	26(4.4)
**Mixed**	10(1.68)	10(1.68)	1(0.16)	7(1.18)	-	5(0.84)	4(0.67)	37(6.2)
**UT**	45(7.58)	30(5.0)	10(1.68)	25(4.21)	-	3(0.5)	32(5.3)	145(24.5)
**Total**	166(28)	142(23.9)	76(12.8)	81(13.7)	3(0.5)	34(5.7)	91(15.3)	593(100)

Approximately 25% (145/593) specimens were untypeable for P and 5.3% (32/593) were untypeable for G. The samples containing more than one type of G strains were 5.7% (34/593) and for P strains were 6% (37/593) strains.

## Discussion

RV is responsible for 1 in 3 cases of severe acute gastroenteritis in children less than 5 years of age hospitalized with diarrhea in urban Pakistani hospitals. The detection rate of 30.5% is lower than the estimated overall Asia burden of 37.5%, though wide variability ranging from 12% in Hong Kong to 56% in Cambodia has been reported [25]–[28]. Our study showed that most cases of RV are in children under the age of 2 years (87%), similar to that described from other Asian settings.

Our study measured the proportion of acute severe watery diarrhea due to RV. The sentinel hospitals, being tertiary care hospitals and referral centers, did not provide services for patients from well-defined catchment areas; hence, incidence rates were not calculated. Acute severe watery diarrhea is affected by other causative agents of diarrhea found in environments of poor water and sanitation. The recently published Global Enterics Multicentric Study (GEMS) in which community primary care sites from Karachi, Pakistan also participated, sheds light on other major causative agents of moderate to severe diarrhea in children (Enterotoxigenic *E. coli*, *Shigella*, cholera, cryptosporidia, and *Aeromonas*); however, it confirmed that RV had the highest attributable fraction in children aged 0 – 11 months, and declined with age [6]. The attributable fraction of acute severe watery diarrhea due to RV was lower compared to our study since RV is more likely to be a causative agent when the case definition of severe acute watery diarrhea is applied as opposed to moderate-to-severe diarrhea considered in GEMS.

RV is characterized by seasonality, causing disease in temperate climates mostly during winter months [29], [30]. RV activity continued all year round like many other tropical Asian countries [31], [32]. While proportional RV peaks did occur in the winter months (November – March), total diarrheal disease burden rose in the summer (May – August). Further, RV negative diarrhea more often presented with fever, vomiting, and had slightly longer duration compared to RV positive diarrhea patients. The greater severity of RV negative diarrhea contrasts with reports from high income countries, and may be due to other serious causes of acute watery diarrhea such as ETEC, *Shigella*, cholera, cryptosporidiosis, and *Aeromonas*, described in the GEMS study [6]. Therefore, severity of symptoms (i.e., the presence of fever and vomiting and relatively longer duration of diarrhea; Vesikari score was not calculated) is not helpful in diagnosing acute gastroenteritis of RV etiology. Genotypic profiling revealed the predominant P strain was P[6] (31.5%) followed by P[8] (20.2%). The G1P[8] combination previously responsible for majority of the childhood diarrhea cases in European countries, was found to be the most prevalent in our study as well but accounted for mere 11.6% of the strains [33], [34]. This strain was found to be the commonest in a single center hospital surveillance study conducted in Faisalabad, Pakistan but was present in only 2.3% of RV positive samples in another single center study in a hospital in Lahore [12], [13]. Community based surveillance data had previously shown G1 and G9 to be almost similar [14]. A small proportion of G12 strains were also seen. The detection of G9 and G12 is in keeping with global, regional and local reports of their emergence [12], [35], [36]. Untypeable strains also constituted a significant portion of the specimens, implying the presence of new strains arising due to mutations which could not be detected with the primers used. Improvement in genetic sequencing and development of new primers will help identify these new strains and reduce the proportion of untypeable strains in the future. While RV vaccines have exhibited cross protection against other strains, natural variability in RV strains and changes following the introduction of the RV vaccines need to be considered when evaluating impact of existing vaccines and development of new vaccines [37]–[39].

Our study's main strengths are that it was a multicenter study, with five different sites across the country and surveillance was conducted for two years. A standardized case definition and methodology were used at all sites to minimize inter-site variability and allow cumulative analysis of data.

There are a few limitations of our study. Only proportions were analyzed and not incidence because catchment areas were not well-defined. We were not able to document in-hospital mortality among enrolled children because children enrolled in the study received prompt medical attention following WHO protocols of hydration. There was potential selection bias towards children with malnutrition who have a higher chance of getting admitted to the hospital compared to normally nourished children. The proportion of untyped strains was high due to lack of resources for further genetic sequencing.

One-third of children under-5 years admitted to hospitals in Pakistan with acute severe watery diarrhea are suffering from RV gastroenteritis. Introduction of RV vaccine of even moderate efficacy in Pakistan can potentially save thousands of admissions and avert RV-related mortality. Monitoring of RV disease burden and strain variability in countries with the greatest RV-related mortality needs to continue to assess the impact of vaccine.
